# Epidemiological Analysis of 5,595 Procedures of Endovascular Correction of Isolated Descending Thoracic Aortic Disease Over 12 Years in the Public Health System in Brazil

**DOI:** 10.6061/clinics/2021/e2890

**Published:** 2021-07-08

**Authors:** Maria Fernanda Cassino Portugal, Marcelo Passos Teivelis, Marcelo Fiorelli Alexandrino da Silva, Alexandre Fioranelli, Claudia Szlejf, Edson Amaro-Júnior, Nelson Wolosker

**Affiliations:** I Hospital Israelita Albert Einstein, Sao Paulo, SP, BR; II Faculdade Israelita de Ciencias da Saude Albert Einstein, Sao Paulo, SP, BR; III Faculdade de Ciencias Medicas da Santa Casa de Sao Paulo, Sao Paulo, SP, BR; IV Faculdade de Medicina FMUSP, Universidade de Sao Paulo, Sao Paulo, SP, BR

**Keywords:** Aortic Aneurysm, Aneurysm Surgery, Aorta Thoracic

## Abstract

**OBJECTIVES:**

In Brazil, descending thoracic aorta disease, including aneurysms and dissections, is managed preferentially by endovascular treatment, owing to its feasibility and good results. In this study, we analyzed endovascular treatment of isolated descending thoracic aortic disease cases in the Brazilian public health system over a 12-year period.

**METHODS:**

Public data from procedures performed from 2008 to 2019 were extracted using web scraping techniques to assess procedure type frequency (elective or urgency), mortality, and governmental costs.

**RESULTS:**

A total of 5,595 procedures were analyzed, the vast majority of which were urgent procedures (61.82% *vs.* 38.18%). In-hospital mortality was lower for elective than for urgent surgeries (4.96 *vs.*10.32% *p*=0.008). An average of R$16,845.86 and R$20,012.04 was paid per elective and emergency procedure, respectively, with no statistical difference (*p*=0.095).

**CONCLUSION:**

Elective procedures were associated with lower mortality than urgent procedures. There was no statistically significant difference between elective and urgent procedures regarding costs.

## INTRODUCTION

Thoracic aortic disease (TAD) encompasses aneurysms and acute aortic syndromes (dissections, intramural hematomas, and penetrating atherosclerotic ulcers). Aneurysms, the most frequent presentation, are localized permanent dilatations covering ≥50% of an artery’s diameter (1-3); the thoracic aortic size thresholds for elective repair indication are determined by the dilation location and underlying etiology (4). Current guidelines recommend surgical repair from a 5.5-cm diameter for idiopathic or sporadic dilatations, and a lower threshold of 5 cm in case of Marfan syndrome or a familial thoracic aortic aneurysm (5). While thoracic aortic aneurysms are typically asymptomatic, acute types usually present with acute chest or back pain (4).

Population studies published in the late 20^th^ century reported an incidence of thoracic aorta aneurysms (TAAs) from 5.9 to 10.4 per 100,000 person-years (6,7) and aortic dissection from 2.9 to 4 per 100,000 person-years (8-11). However, improvements in imaging technology and a higher quotient of older patients have affected these rates, with reported rates as high as 24.4 per 100,000 persons in 2016 (12). In autopsies, the prevalence of descending TAAs was estimated at 3% (4). Specifically, one retrospective analysis of tomographs in Brazil found a 1.08% TAA prevalence (13).

Therapeutic alternatives vary according to the aortic disease location. When the aortic root, ascending aorta, and arch are affected, repair is usually performed via median sternotomy with cardiopulmonary bypass (4). For isolated descending TAD (I-DTAD), options include open surgical repair and endovascular treatment (TEVAR). Since a technological transition in Brazil was first observed in the early 2000s (14), most groups have focused on endovascular repair for TAD, with open surgeries representing a small portion of all cases.

Most information on TAA incidence and outcomes is based on noncurrent studies with limited sample sizes or time intervals and results from specialized centers or predating the widespread use of modern imaging modalities (15). However, real-life scenario information is of higher and paramount importance in guiding public health resources.

To the best of our knowledge, only one large-scale study, which evaluated procedures performed by the public health system from 2008 to 2018, has been published in Brazil with respect to I-DTAD management; however, it was focused on the city of São Paulo (16). For this reason, in this study, we aimed to evaluate the endovascular management of I-DTAD, encompassing all procedures performed in the public health system from 2008 to 2019, assessing procedure type frequency (elective or urgency), mortality, and governmental costs. This is the first study of its type published in Brazil encompassing nationwide data.

## METHODS

This study was approved by the institution’s ethics committee under protocol 35826320.2.0000.0071.

Public data concerning endovascular surgeries for I-DTAD repair performed from 2008 to 2019 were extracted from the DATASUS portal, which is a digital platform, although the Brazilian government provides open data relating to procedures performed under the public health system in accredited hospitals. Institutional accreditation is a prerequisite for receiving government payment for the performed procedures. All the data were appropriately de-identified. Our search was conducted on November 15, 2020. We did not include thoracoabdominal procedures.

Data referring to endovascular treatment procedures for I-DTAD from 2008 to 2019 were selected from the platform, along with information regarding procedure technique, in-hospital mortality, and cost. Reported values refer to hospitalization costs, including those for surgical devices. Procedures were identified using the Brazilian Public Health System Protocol of Procedures and Medicines code for endovascular aneurysm or thoracic aortic dissection repair with a straight/conical stent graft (code: 04.06.04.017-6). Unfortunately, the system coding for both the diagnosis (International Classification of Diseases, Tenth Revision) and treatment procedure (procedural coding) are superimposed for aneurysms and dissections, rendering the separation of cases infeasible. It is also not possible to exclude the codes for congenital diseases, which should account for a very small proportion of the evaluated cases.

The selected cases were classified as either elective, which are patients undergoing surgery on an elective basis, or urgent, which are patients needing urgent hospital admissions.

An automated web scraping method was used for data collection. The codes employed were programmed in Python (v2.7.13; Beaverton, OR, USA) using the Windows 10 Single Language operating system. Field selection in the DATASUS platform and posterior table adjustment were performed using Selenium WebDriver packages (v3.1.8; Selenium HQ, various contributors worldwide) and pandas (v2.7.13; Lambda Foundry, Inc. and PyData Development Team, NY, USA). All data were organized into Microsoft^®^ Office Excel 2016 (v16.0.4456.1003; Redmond, WA, USA) spreadsheets after collection and treatment.

### Statistical analysis

Trends in the distribution of procedure techniques were evaluated using the chi-square test. Mortality rates and average costs of the two groups were compared using the Mann-Whitney test.

The level of statistical significance was set at *p*<0.05.

## RESULTS

A total of 5,595 endovascular procedures were performed by the Brazilian public health system from 2008 to 2019 for I-DTAD correction.

Table 1 depicts the number and frequency distributions of the procedures. Urgency procedures were more frequent in all evaluated years (61.82% *vs.* 38.18%), and a trend test indicated an inclination towards an increase in urgent procedures and a decrease in elective procedures throughout the years (*p*<0.001).

Mortality per procedure type per geographic region is shown in Table 2. There were 432 in-hospital deaths (general mortality rate, 7.72%). In-hospital mortality was lower for elective procedures (4.96% *vs.* 10.32% *p*=0.008).

The government costs are shown in Table 3. A total of R$102,005,373.62 was paid for all procedures. Elective and urgent procedures were paid an average of R$16,845.86 and R$20,012.04 (18.79% higher), respectively, which was not a statistically significant difference (*p*=0.095).

## DISCUSSION

An automated method using web scraping codes was devised by our Institution’s Informatics Department for data gathering, as a manual method, although technically feasible, would have been rather burdensome. All information was retrieved from the public governmental platform DATASUS. However, information sourced from an administrative database instead of medical records is not exempt from coding errors. Additionally, the coding system may permit a certain ambiguity of codes for procedures employed for different diseases, and since the diagnosis cannot be tracked from the de-identified database, exclusion of unwanted cases is not possible. However, because of the bulk of cases, only a small portion of collected data was incorrectly considered.

Brazil had an estimated 211,755,692 inhabitants in 2020 (17). As of June 2020, over 75% of them have been solely dependent on the tax-funded governmental system called Sistema Único de Saúde (SUS), whereas 24.1% have benefited from private supplementary healthcare, either individually financed or maintained by an employer (18).

The worldwide incidence of thoracic aortic aneurysms is estimated at 5.6 to 10.4 cases per 100,000 patient-years (7,19). Although large population studies are scarce, a nationwide analysis in Sweden found an annual TAD incidence of 16.3 per 100,000 males and 9.1 per 100,000 females (15). In a survey of the Manitoba province in Canada, the age-standardized TAD incidence increased from 16.8 per 100,000 persons in 1998 to 24.4 per 100,000 persons in 2016 (12). If the worldwide estimation is applied to the approximate population dependent on the Public Health System in Brazil in the year 2020 (17,18), 9,000-16,715 new TAAs are expected annually. However, the yearly average of operated patients was 466.25, representing approximately 2.78-5.18% of expected diagnostics. Assessment of the population of São Paulo showed that 20-40% of expected TAAs were covered under the yearly average rates for treatment (16). Despite the inability to establish the proportion of diagnosed cases with indisputable surgical indications from the database, as well as the absence of exact data regarding the mortality rates of unoperated I-DTAD in Brazil, the determined intervention rate is lower than expected.

This evaluation is relatively limited because open surgeries for TAD were not included in this analysis. Although the benefit of TEVAR cannot be definitively established because of the lack of randomized controlled trials (20-24), the endovascular technique is a less complex method with good results in terms of early mortality and complications, whereas conventional repair usually requires a more complex intraoperative and postoperative hospital structure (4). For this reason, since the late 1990s and the early 2000s, most groups in Brazil who treat I-DTAD focus on endovascular repair procedures, which are fully covered by the public health system.

The operation delay for cases in which surgical correction is unequivocally indicated may incur rupture and death (23). It is reasonable, therefore, to assume that well-structured systems should present a majority of elective procedures, as opposed to urgent ones. This was not the case in the Brazilian territory, as urgent procedures have been more common (61.82%) and more frequent annually, with a growing tendency to surpass elective procedures (Table 1). The literature suggests that elective procedure rates worldwide vary between 68.4% and 80% (25-28). The inversion of this standard in Brazil may indicate the need for greater investment in public care regarding TAD screening in patients at a greater risk (for instance, those with abdominal aortic aneurysms or smokers aged >50 years) to try and enhance detection while the patient remains asymptomatic. Conversely, in selected cases, patients may be considered unfit for elective surgery at diagnosis, being operated only if symptomatic in an urgent scenario. Because our information is de-identified, thereby indicating a void of such particularities, the proportion of cases in which this may have happened is unknown.

The in-hospital mortality rate was higher for the urgent procedures (10.32% *vs.* 4.96%, *p*=0.008), according to data from the North American National Inpatient Sample, in which elective surgery was protective against inpatient mortality (OR, 0.76; 95% CI, 0.58-0.99; *p*=0.042) (25). The elective group mortality rate (4.96%) was similar to that in a series of other studies (29), although some included more complex aneurysms in the study samples, reinforcing the advantage of operating while patients remain asymptomatic.

Governmental investment was not statistically different between groups (R$16,845.86 per elective case *vs.* R$20,012.04 per urgency case, *p*=0.095). However, this analysis is based on the SUS reimbursement values, which are often subpar with the actual hospital procedural cost. In addition to the fact that early mortality with short hospital stays may homogenize values between groups, the cost analysis may be somewhat undermined.

Although some authors found that endovascular correction was advantageous in terms of quality-adjusted life-years gain (30), a study by Arnaoutakis et al. conducted at the Johns Hopkins Hospital reported that TEVAR did not significantly reduce overall hospital charges, specifically because of device costs (31).

### Limitations

As is common with other retrospective analyses, our study is limited by the loss of patient information. Because the database only compiled data from accredited hospitals listed in the DATASUS databank, data loss certainly occurred, especially in the emergency scenario. Additionally, a fraction of procedures for aortic trauma, rather than atherosclerotic degeneration, may have been inadvertently included.

Since very few institutions in Brazil focus on conventional TAD repair, the frequency of these procedures bore no analytical importance, hence they were not included.

Several other limitations were derived as consequences of the anonymized databank information. First, patient follow-up is not possible, and all mortality data referred exclusively to in-hospital deaths, rendering this study unable to contribute information with respect to long-term mortality rates. Second, we were also unable to provide input regarding the need for reinterventions (which are relatively more frequent in the context of endovascular procedures).

Despite its limitations, this study provides a comprehensive analysis of the public health systems management of TAD in one of the largest low- and middle-income countries of the world, encompassing a 12-year interval and over 5,595 procedures, granting a representational assessment of a real-world sample of I-DTAD patients.

## CONCLUSION

In the Brazilian Public Health System, a total of 5,595 procedures were performed for the correction of I-DTAD over a period of 12 years. Urgent procedures were vastly more frequent and incurred a higher mortality rate than elective procedures. There was no significant difference in reimbursements between urgent and elective procedures.

## AUTHOR CONTRIBUTIONS

Portugal MF was responsible for the manuscript composition, critical revision, manuscript acceptance, and agreement to accountability. Teivelis MP and Wolosker N were responsible for the manuscript conception and composition, critical revision, manuscript acceptance, and agreement to accountability. Silva MF and Amaro-Júnior E were responsible for the manuscript conception, data collection, manuscript acceptance, and agreement to accountability. Fioranelli A was responsible for the manuscript conception, critical revision, manuscript acceptance, and agreement to accountability. Szlejf C was responsible for the manuscript conception, data collection, manuscript acceptance, and agreement to accountability.

## Figures and Tables

**Table 1:** Absolute and relative frequency of elective and urgent procedures for thoracic aorta aneurysms from 2008 to 2019.

	Elective		Urgency			*p* *
	n	%	n	%	Total	
2008	160	43.72	206	56.28	366	<0.001
2009	195	44.42	244	55.58	439	
2010	204	45.03	249	54.97	453	
2011	168	41.38	238	58.62	406	
2012	171	40.43	252	59.57	423	
2013	239	45.01	292	54.99	531	
2014	223	42.72	299	57.28	522	
2015	183	35.81	328	64.19	511	
2016	188	36.94	321	63.06	509	
2017	145	27.72	378	72.28	523	
2018	148	29.54	353	70.46	501	
2019	112	27.25	299	72.75	411	
Total	2,136	38.18	3,459	61.82	5,595	

* trend chi-square test.

**Table 2:** Absolute and relative mortality per geographic region by procedure type.

Geographic Region	Total			Procedure Type					
				Elective			Urgency		
	Procedures	Mortality n	(%)	Procedures	Mortality n	(%)	Procedures	Mortality n	(%)
North	142	8	5.63	84	3	3.57	58	5	8.62
Northeast	594	34	5.72	377	13	3.45	174	21	12.07
Southeast	2,982	248	8.32	1,275	80	6.27	1,563	168	10.75
South	1,527	110	7.20	329	5	1.52	1,103	105	9.52
Centre-West	350	32	9.14	71	5	7.04	262	27	10.31
Total	5,595	432	7.72	2,136	106	4.96	3,160	326	10.32
*p* *								0.008	

* Mortality in those who underwent urgent procedures was statistically higher than in those who underwent elective procedures (*p*=0.008), Mann-Whitney test.

**Table 3:** Values passed by SUS in Brazilian Reals per geographic region by procedure type.

Total Amount	Amount Paid per Procedure		Average Amount Paid per Patient	
	Elective Procedure	Urgency Surgery	Elective Procedure	Urgency Surgery
2,240,441.08	1,306,248.82	934,192.26	15,550.58	16,106.76
10,271,277.36	6,342,584.26	3,928,693.10	16,823.83	22,578.70
54,927,504.58	22,315,127.27	32,612,377.31	17,502.06	20,865.24
28,056,465.39	5,591,985.04	22,464,480.35	16,996.92	20,366.71
6,509,685.21	1,232,270.07	5,277,415.14	17,355.92	20,142.81
102,005,373.62	36,788,215.46	65,217,158.16	16,845.86	20,012.04
*p* *			*p*=0.095	

* Cost per patient was not significantly different between both groups (*p*=0.095), Mann-Whitney test.

SUS, Sistema Único de Saúde.

## References

[1] de Almeida Mendes C, de Arruda Martins A, Teivelis MP, Kuzniec S, Varella AY, Wolosker N (2017). Carbon Dioxide as Contrast Medium to Guide Endovascular Aortic Aneurysm Repair. Ann Vasc Surg.

[2] Johnston KW, Rutherford RB, Tilson MD, Shah DM, Hollier L, Stanley JC (1991). Suggested standards for reporting on arterial aneurysms. Subcommittee on Reporting Standards for Arterial Aneurysms, Ad Hoc Committee on Reporting Standards, Society for Vascular Surgery and North American Chapter, International Society for Cardiovascular Surgery. J Vasc Surg.

[3] Puech-Leão P, Kauffman P, Wolosker N, Anacleto AM (1998). Endovascular grafting of a popliteal aneurysm using the saphenous vein. J Endovasc Surg.

[4] Dudzinski DM, Isselbacher EM (2015). Diagnosis and Management of Thoracic Aortic Disease. Curr Cardiol Rep.

[5] Hiratzka LF, Bakris GL, Beckman JA, Bersin RM, Carr VF, Casey DE (2010). 2010 ACCF/AHA/AATS/ACR/ASA/SCA/SCAI/SIR/STS/SVM guidelines for the diagnosis and management of patients with Thoracic Aortic Disease: a report of the American College of Cardiology Foundation/American Heart Association Task Force on Practice Guidelines. Circulation.

[6] Bickerstaff LK, Pairolero PC, Hollier LH, Melton LJ, Van Peenen HJ, Cherry KJ (1982). Thoracic aortic aneurysms: a population-based study. Surgery.

[7] Clouse WD, Hallett JW, Schaff HV, Gayari MM, Ilstrup DM, Melton LJ (1998). Improved prognosis of thoracic aortic aneurysms: a population-based study. JAMA.

[8] Mészáros I, Mórocz J, Szlávi J, Schmidt J, Tornóci L, Nagy L (2000). Epidemiology and clinicopathology of aortic dissection. Chest.

[9] Clouse WD, Hallett JW, Schaff HV, Spittell PC, Rowland CM, Ilstrup DM (2004). Acute aortic dissection: population-based incidence compared with degenerative aortic aneurysm rupture. Mayo Clin Proc.

[10] Sato F, Kitamura T, Kongo M, Okinaka T, Onishi K, Ito M (2005). Newly diagnosed acute aortic dissection: characteristics, treatment modifications, and outcomes. Int Hear J.

[11] Yu HY, Chen YS, Huang SC, Wang SS, Lin FY (2004). Late outcome of patients with aortic dissection: study of a national database. Eur J Cardiothorac.

[12] Lodewyks CL, Prior HJ, Hiebert BM, Nickel NC, Yamashita MH, Ouzounian M (2020). A Province-Wide Analysis of the Epidemiology of Thoracic Aortic Disease: Incidence Is Increasing in a Sex-Specific Way. Can J Cardiol.

[13] Góes AMO Junior, Mascarenhas Bĺ, Rodrigues SC, de Andrade MC, Franco RSM (2016). Achados incidentais de aneurismas torácicos e abdominais. J Vasc Bras.

[14] Puech-Leão P, Wolosker N, Zerati AE, Nascimento LD (2011). Impact of endovascular technique in vascular surgery training at a large university hospital in Brazil. J Surg Educ.

[15] Olsson C, Thelin S, Ståhle E, Ekbom A, Granath F (2006). Thoracic aortic aneurysm and dissection: increasing prevalence and improved outcomes reported in a nationwide population-based study of more than 14,000 cases from 1987 to 2002. Circulation.

[16] Portugal MFC, Teivelis MP, Silva MFAD, Stabellini N, Fioranelli A, Szlejf C (2021). Endovascular correction of isolated descending thoracic aortic disease: a descriptive analysis of 1,344 procedures over 10 years in the public health system of São Paulo. Clinics (Sao Paulo).

[17] IBGE - Instituto Brasileiro de Geografia e Estatística Estimativas Populacionais. São Paulo, Rio de Janeiro, 2020. https://www.ibge.gov.br/estatisticas/sociais/populacao/9103-estimativas-de-populacao.html?=&t=o-que-e.

[18] Agência Nacional de Saúde Suplementar do Brasil (2020). Sala de Situação.

[19] Howard DP, Banerjee A, Fairhead JF, Perkins J, Silver LE, Rothwell PM (2013). Population-based study of incidence and outcome of acute aortic dissection and premorbid risk factor control: 10-year results from the Oxford Vascular Study. Circulation.

[20] Heijmen RH, Deblier IG, Moll FL, Dossche KM, van den Berg JC, Overtoom TT (2002). Endovascular stent-grafting for descending thoracic aortic aneurysms. Eur J Cardiothorac Surg.

[21] Bortone AS, De Cillis E, D’Agostino D, de Luca Tupputi Schinosa L (2004). Endovascular treatment of thoracic aortic disease: four years of experience. Circulation.

[22] Walsh SR, Tang TY, Sadat U, Naik J, Gaunt ME, Boyle JR (2008). Endovascular stenting versus open surgery for thoracic aortic disease: systematic review and meta-analysis of perioperative results. J Vasc Surg.

[23] Abraha I, Romagnoli C, Montedori A, Cirocchi R (2016). Thoracic stent graft versus surgery for thoracic aneurysm. Cochrane Database Syst Rev.

[24] Cheng D, Martin J, Shennib H, Dunning J, Muneretto C, Schueler S (2010). Endovascular aortic repair versus open surgical repair for descending thoracic aortic disease a systematic review and meta-analysis of comparative studies. J Am Coll Cardiol.

[25] Wang GJ, Jackson BM, Foley PJ, Damrauer SM, Goodney PP, Kelz RR (2018). National trends in admissions, repair, and mortality for thoracic aortic aneurysm and type B dissection in the National Inpatient Sample. J Vasc Surg.

[26] Knowles M, Murphy EH, Dimaio JM, Modrall JG, Timaran CH, Jessen ME (2011). The effects of operative indication and urgency of intervention on patient outcomes after thoracic aortic endografting. J Vasc Surg.

[27] Dick F, Hinder D, Immer FF, Savolainen H, Do DD, Carrel TP (2009). Thoracic endovascular aortic repair: impact of urgency on outcome and quality of life. Eur J Cardiothorac Surg.

[28] O’Donnell TFX, Patel VI, Deery SE, Li C, Swerdlow NJ, Liang P (2019). The state of complex endovascular abdominal aortic aneurysm repairs in the Vascular Quality Initiative. J Vasc Surg.

[29] Knepper J, Upchurch GR (2010). A review of clinical trials and registries in descending thoracic aortic aneurysms. Semin Vasc Surg.

[30] Luebke T, Brunkwall J (2014). Cost-effectiveness of endovascular versus open repair of acute complicated type B aortic dissections. J Vasc Surg.

[31] Arnaoutakis GJ, Hundt JA, Shah AS, Cameron DE, Black JH 3rd (2011). Comparative analysis of hospital costs of open and endovascular thoracic aortic repair. Vasc Endovascular Surg.

